# Genetic Analysis of *MECP2* Gene in Iranian Patients with Rett Syndrome

**Published:** 2019

**Authors:** Jafar NASIRI, Mansoor SALEHI, Majid HOSSEINZADEH, Mahdi ZAMANI, Shirin FATTAHPOUR, Omid ARYANI, Esmat FAZEL NAJAFABADI, Maryam JABARZADEH, Sara ASADI, Tahereh GHOLAMREZAPOUR, Maryam SEDGHI, Fatemeh GHORBANI

**Affiliations:** 1 Department of Paediatric Neurology, Faculty of Medicine, Child Growth and Development Research Center, Isfahan University of Medical Sciences, Isfahan, Iran; 2 Medical Genetics Laboratory, Alzahra University Hospital, Isfahan University of Medical Sciences, Isfahan, Iran; 3 Department of Medical Genetics, School of Medicine, International Campus, Tehran University of Medical Sciences, Tehran, Iran; 4 Department of Neuroscience, Iran University of Medical Sciences, Tehran, Iran

**Keywords:** Rett syndrome, MECP2 mutation, Direct sequencing, Iran

## Abstract

**Objectives:**

Rett syndrome is an X linked dominant neurodevelopmental disorder which almost exclusively affects females. The syndrome is usually caused by mutations in *MECP2* gene, which is a nuclear protein that selectively binds CpG dinucleotides in the genome.

**Materials & Methods:**

To provide further insights into the distribution of mutations in *MECP2* gene, we investigated 24 females with clinical characters of Rett syndrome referred to Alzahra University Hospital in Isfahan, Iran during 2015-2017. We sequenced the entire *MECP2* coding region and splice sites for detection of point mutations in this gene. Freely available programs including JALVIEW, SIFT, and PolyPhen were used to find out the damaging effects of unknown mutations.

**Results:**

Direct sequencing revealed *MECP2* mutations in 13 of the 24 patients. We identified in 13 patients, 10 different mutations in *MECP2* gene. Three of these mutations have not been reported elsewhere and are most likely pathogenic.

**Conclusion:**

Defects in *MECP2* gene play an important role in pathogenesis of Rett syndrome. Mutations in *MECP2* gene can be found in the majority of Iranian RTT patients. We failed to identify mutations in *MECP2* gene in 46% of our patients. For these patients, further molecular analysis might be necessary.

## Introduction

Rett syndrome, is a severe X linked dominant neurodevelopmental disorder that predominantly affects females, and has been considered lethal to males (1, 2). 

Typically, this syndrome is characterized by apparently normal development during the first 6 months of life, followed by rapid deterioration with regression of social, motor, and communication skills (1). Clinical features also include microcephaly, seizures, stereotypical hand movements, scoliosis, ataxia, intellectual disability, and little or no verbal skills (3). Rett syndrome is usually due to mutations in the *MECP2* gene, located on chromosome X (Xq28) and encodes a methyl-CpG binding protein 2 (*MECP2*) (1). *MECP2* is a nuclear protein that selectively binds CpG dinucleotides in the mammalian genome and may act as a silencer of gene expression interacting with other proteins such as histone deacetylase complex and the transcriptional corepressor sin3A (4).

*MECP2 *gene has two protein isoform. Both isoforms contain five major domains: N-terminal domain (NTD), Methyl binding domain (MBD), Inter-Domain (ID), Transcription repression domain (TRD) and C-terminal domain (CTD) (5). The majority of Rett patients have mutations in exons 3 or 4, which encode the MECP2 functional domains, the MBD and the TRD domain, respectively (1).

To our knowledge, so far there has been no comprehensive study on Rett syndrome in Iran. To provide further insights into the spectrum and distribution of mutations in *MECP2* gene, we performed sequencing of the entire *MECP2* coding regions and splice sites in 24 female cases with clinical characteristics of Rett syndrome from the Iranian population.

## Materials and Methods

Molecular genetic analysis of *MECP2* gene was carried out in 24 sporadic Iranian female patients with Rett syndrome referred to Alzahra University Hospital in Isfahan, Iran during 2015-2017. 

This study was approved by the Ethics Committee of Isfahan University of Medical Sciences and consent forms were obtained from all the patients’ parents.

Clinical diagnosis was according to the diagnostic criteria (6). The patients had at least five of the necessary criteria proposed by the Rett syndrome Diagnostic Criteria Work Group (7) (Table 1). Clinical details of each patient were recorded by neurologists (Table 2).

We prepared genomic DNA from peripheral blood leukocytes of patients by the standard salting-out method (8). PCR was performed for exons 1, 2, 3, and 4 using primer pairs reported in Table 3. For exons 2, 3 and 4, PCR reactions were performed with Taq DNA polymerase kit (Feldan, Germany) in a final volume of 50 µl, using 5 µl band sharpener, 5 µl 10x Taq Buffer, 1 µl dNTP mix (10 mM), 0.4 µl Taq DNA polymerase (5 U/μl), 2 µl of forward and reverse primer (10 pmole/μl) and 200 ng of DNA. For the amplification of exon 1, a GC-rich amplicon difficult to amplify, the optimization of the PCR protocol implied the use of the same components plus 5 µl of DMSO (5 M). Cycling parameters were 94 °C for 5 min, followed by 30 cycles of 94 °C for 1 min, 63 °C for 1 min, 72 °C for 1 min and a final step of 72 °C for 10 min. All PCR products were visualized on 1% agarose gel and were sequenced on an ABI 3130 sequencer (Applied Biosystems, United Kingdom) with a 36-cm capillary array and POP-4 polymer (Applied Biosystems). All the patients were sequenced for all five fragments. The obtained nucleotide sequences were compared to the reference sequence in Gene Bank (Ref Seq NC_000023.11) using BLAST and sequence variation was confirmed on both strands. All the sequence variations detected in this study were confirmed by a new sequencing performed from a newly amplified sample. In silico analysis was performed for novel mutations.

## Results

Direct sequencing revealed *MECP2* mutations in 13 of the 24 patients. All the detected mutations were compared with RettBASE: *MECP2* variation database (http://mecp2.chw.edu.au/). The mutations include four missense mutations (A73D, R306C, T158M and R106W), two nonsense mutations (R168X and R255X), three deletions (R270fs, K286fs, K233fs) and one complex deletion/insertion (G237fs) based on the NM_004992.3 transcript (Table 4). Although the spectrum of mutations is very heterogeneous, mutations mainly occur in exon 4, which points to mutational hotspots in *MECP2* gene. 

**Figure 1:**
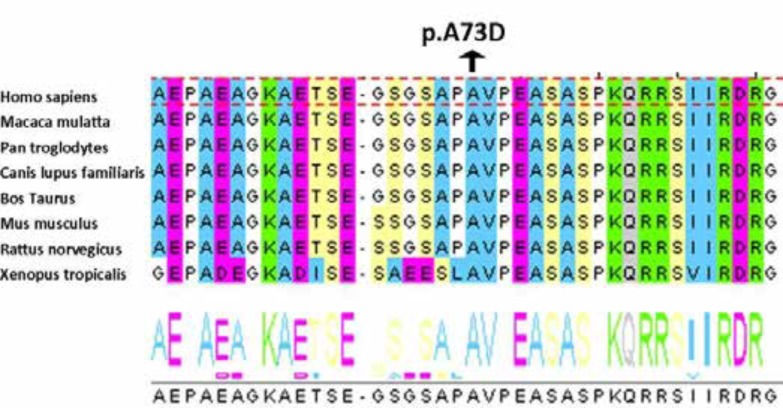
Analysis by Jalview showed that amino acid 73 is conserved in mammals

In order to find out the functional consequences of this new missense mutation, we performed in silico analyses with JALVIEW, SIFT (http://sift.jcvi.org/) and PolyPhen (http://genetics.bwh.harvard.edu/pph2/). c.218 C>A is pathogenic. Multiple alignments of the sequences using Jalview showed that the amino acid position in exon 3 is physicochemically conserved in mammals (Figure 1).

## Discussion

With the discovery of mutations in *MECP2* gene, RTT became the first human disease to be caused by mutations in a gene encoding a factor that has role in the epigenetic silencing machinery (9).

In our study, three of the thirteen patients with *MECP2* mutations had one of the nonsense mutations reported by Wan et al (9). Two of them had the R168X mutation, which encodes a truncated *MECP2* protein containing only the MBD and lacking the TRD, the nuclear localization signal and the C-terminal region (11). Since the R168X mutation is within the last exon, between the MBD and TRD, it is not expected to cause nonsense-mediated decay; therefore, the truncated form of the protein may retain its ability to bind to 5-methyl CpG. However, as the nuclear localization signal (NLS) is within the TRD, the truncated protein lacking the NLS, may stay in the cytoplasm (9, 12). The other patient had the c.763C > T common mutation which substitutes the conserved arginine 255 (CGA) to a stop codon (TGA) creating a truncated *MECP2* protein. This p.R255X nonsense mutation is located in TRD-NLS of *MECP2 *protein (13). Six patients in this study had a missense mutation. The missense mutation R306C was found in 3 out of 13 patients (23%). The R306C mutation is located in the C-terminal part of the TRD and is the first missense mutation identified in the TRD (9). Amino acids 269-309 of *MECP2* are necessary for binding to the NCoR/SMRT co-repressor complex and loss of this connection gives rise to RTT. The R306C mutation, abolish the association between the NCoR/SMRT complex and *MECP2 *protein, which can cause Rett syndrome (14). 

**Table 1:** Diagnostic criteria for Rett syndrome (14)

**Necessary criteria**
(1)Apparently normal prenatal and perinatal period (2)Apparently normal psychomotor development through the first 6 months (3)Normal head circumference at birth (4)Deceleration of head growth between the ages of 5 months and 4 yr (5)Loss of acquired purposeful hand skills between the ages of 6 and 30 months, temporally associated with communication dysfunction and social withdrawal (6)Development of severely impaired expressive and receptive language, and presence of apparent severe psychomotor retardation. (7)Stereotypic hand movements such as hand wringing/squeezing, clapping/tapping, mouthing and washing/rubbing automatisms appearing after purposeful hand skills are lost (8)Appearance of gait apraxia and truncal apraxia/ataxia between ages 1 and 4 yr of age (9)Diagnosis tentative until 2-5 yr of age

**Table 2:** Clinical features of Rett patients with MECP2 mutations. Clinical features were assigned +, - or 0 which is consistent with positive, negative and not identified respectively

**Patient ID**	**Current age**	**Type**	**Clinical symptoms**												
			**Postnatal microcephaly**	**Early normal psychomotor development**	**Facial dysmorphism**	**Ability to walk**	**Impaired speech**	**loss of hand function**	**Stereotypic hand movements**	**Diminished response to pain**	**Psychomotor retardation**	**Scoliosis**	**Bruxism**	**Cold extremities**	**Seizure**
1	3 year	Classical	+	+	-	-	+	+	+	+	+	-	+	+	+
2	11 year	Classical	+	+	-	-	+	+	+	0	+	-	-	-	-
3	7 year	Atypical	+	+	+	-	+	+	+	-	-	+	+	+	+
4	5 year	Classical	-	+	-	-	+	-	+	+	+	-	+	-	-
5	3.5 year	Classical	-	+	-	-	+	+	+	+	+	+	-	-	-
6	22 month	Classical	-	+	-	-	+	+	+	+	+	+	+	+	-
7	8 year	Atypical	+	+	-	+	-	-	+	-	-	+	+	-	+
8	4 year	Classical	+	+	+	-	+	-	+	+	+	+	+	-	-
9	6 year	Atypical	+	-	-	+	-	-	+	-	-	+	+	+	+
10	5.5 year	Classical	+	+	-	-	+	+	+	+	+	-	-	+	-
11	3.5 year	Classical	+	-	-	-	+	+	+	-	+	-	+	-	+
12	10 year	Classical	+	-	+	+	+	-	+	-	+	+	+	+	+
13	6 year	Atypical	+	-	-	+	-	-	+	-	-	+	+	+	+

**Table 3:** The primers and PCR conditions designed for the analysis of the MECP2 gene

Exon		Fragment		Primers	Product size(bp)	Tm(˚C)
Exon 1		Forward		CAAGCCTAGGCCTTCACTTGCC	610 bp	66 ˚C
		Reverse		CATCCGCCAGCCGTGTCG		
Exon 2		Forward		AGTGTGTTTATCTTCAAAATGT	376 bp	63 ˚C
		Reverse		GTTATGTCTTTAGTCTTTGGG		
Exon 3		Forward		CTTGCATGTGGTGGGGGTC	590 bp	63 ˚C
		Reverse		AGTCATTTCAAGCACACCTGGTC		
Exon 4	Exon 4a		Forward	GTTCAATAGTAACGTTTGTCAGAGC	841 bp	63 ˚C
			Reverse	TGGTGGTGCTCCTTCTTGG		
	Exon 4b		Forward	CTGGGCGGAAAAGCAAGGAGAG	545 bp	63 ˚C
			Reverse	GTGATTTCAGTTAATCGGGAAGCTTTG		

**Table 4:** Identified mutations of MECP2 gene

**Patient**	**Mutation in CDNA**	**Mutation in amino acid**	**exon**	**Domain**	**Type of mutation**	**Reference**
1	c.218 C>A	p.A73D	3	NTD	Missense	This study
2	c. 697-701del	p.K233fs	4	TRD	Frame shift	This study
3	c.502 C>T	p.R168X	4	ID	Nonsense	Hoffbuhr et al. (2001)
4	c. 856-859 del AAAG	p.K286fs	4	TRD	Frame shift	Hoffbuhr et al. (2001)
5	c.916C>T	p.R306C	4	TRD	Missense	Wan et al. (1999)
6	c.316C>T	p.R106W	3	MBD	Missense	Amir et al. (1999)
7	c.709-751delinsAAG	p.G237fs	4	TRD	Frameshift	This study
8	c.763C>T	p.R255X	4	TRD	Nonsense	Amir et al. (1999);
9	c.502C>T	p.R168X	4	ID	Nonsense	
10	c.916C>T	p.R306C	4	TRD	Missense	
11	c.473C>T	p.T158M	4	MBD	Missense	Amir et al. (1999)
12	c.916C>T	p.R306C	4	TRD	Missense	
13	c.808delC	p.R270fs	4	TRD	Frameshift	Hoffbuhr et al. (2001)

The other missense mutation R106W causes an arginine to tryptophan amino acid change in the MBD domain. This amino acid substitution may reduce the initial binding to methylated DNA by more than 100-fold and lacks the ability to localize to heterochromatin domains and to repress transcription (15). This mutation is among the eight common mutations in *MECP2* gene, which causes a full RTT phenotype in girls (1, 12, 16). The other patient had the T158M missense mutation which substitutes threonine, a hydrophobic amino acid, with a polar amino acid, methionine in the MBD of *MECP2* protein. This change may affect the C-terminal stretch of MBD, thereby interfering with its function. *MECP2* proteins with T158M mutation in MBD have only a two-fold decrease for methylated DNA. 

Four of our studied cases had a deletion or/and insertion, which two of them have been reported previously and two are possibly novel mutations. The first case had a small deletion of four bases AAAG at position 856-859 in exon four of *MECP2* gene. This deletion leads to the K286 frameshift mutation and a premature stop codon. This premature stop codon leads to the synthesis of a truncated *MECP2* protein. Since this mutation is located in the TRD, it will probably affect the transcriptional repression activity of the *MECP2* protein (17). The second case had a single C base deletion at position 808 in exon 4, which causes a frameshift mutation in the TRD of the protein. In this study, we identified 3 novel mutations. The first one had a small deletion of five bases (AAGGC) in position 696. In the second novel mutation (c.709_751delinsAAG), 42 nucleotides were deleted and 3 nucleotides were inserted. Both of these create a frameshift mutation in the TRD of *MECP2* protein. Since these mutations create truncated protein, they are most likely pathogenic.

The third novel mutation identified in this study was a missense mutation. The A73D mutation substitutes alanine, a non-polar amino acid, with a polar amino acid, aspartic acid in the NTD of *MECP2* protein. In order to assess the pathogenesis of this mutation, DNA samples from both parents were analyzed. None of the parents carried this mutation; therefore this missense mutation is a non-polymorphic variation and is most likely pathogenic.

So far, very few studies have been conducted on Rett syndrome in Iran. The existing reports were limited to the clinical signs in a few patients and a mutation in MECP2 in one patient with Rett syndrome (18-20). For the first time in Iran, we investigated 24 females with clinical characters of Rett syndrome from the Iranian population. We sequenced the entire *MECP2* coding region and splice sites and identified in 13 out of 24 unrelated patients, 10 different mutations. 23.8% of patients with clinical characteristics of Rett syndrome had mutations in *MECP2*. Mutation detection rate varied among different studies which was between 23-90% (4, 9, 10). In this study, 54% of patients had *MECP2* mutations, which is similar to another study (9). Among the molecular defects reported in this study, four are missense mutations, two are nonsense mutations, three are deletions and one is a complex deletion/insertion. Mutations mainly occur in exon 4 and the multiple recurrences of R168X (two times) and R306C (three times), points to true mutational hotspots. These two recurring mutations account for 38% (5 of 13) of all mutation-positive cases. We described three novel mutations not reported previously and are most likely pathogenic. 

To date, more than 250 mutations have been identified in *MECP2*, although eight specific mutations (R106W, P152R, T158M, R168X, R255X, R270X, R288X, and R306C) are found in more than 60% of individuals with RTT (21, 22). Most of the mutations are C>T transitions, which are located in the MBD, TRD or ID. The high frequency of cytosine to thymidine transitions at CpG dinucleotides suggests that deamination of methylated cytosine is a prevalent cause of Rett syndrome (23). We have screened only for mutations in the coding region, and the promoter region was not screened, nor was DNA rearrangements or deletions. Even more, the conserved regions of 3´UTR suggest that these sequences are important for post-transcriptional regulation of *MECP2*. It is acceptable that mutations in the 3´ UTR and the promoter region or maybe DNA rearrangements or deletions of *MECP2* might be the underlying cause of Rett syndrome in those patients without any identified mutations (4, 24). Since not all patients studied so far carry mutations in *MECP2* gene, it is possible that this disorder is genetically heterogeneous. Although MECP2 plays a key role in Rett syndrome, other genes that might interact with MECP2 may also contribute to RTT development (25). Mutations within the CDKL5 and NTNG1 gene have been reported in some patients with clinical characteristics that overlap with RETT syndrome (26). Recent reports have found another gene, FOXG1, to be highly associated with Rett syndrome (27). In our analysis, we failed to detect *MECP2* mutations in 11 of the patients clinically classified as Rett cases. These cases have to be analyzed for mutations in other regions of *MECP2* or in other candidate genes.

Although the number of patients with identified *MECP2* mutations was not enough for statistical analysis, we investigated a genotype-phenotype correlation. We first focused on patients with different *MECP2* mutations and tried to find a genotype-phenotype correlation, taking in to account the type of mutation and the position. An increase in phenotype severity was reported in nonsense mutations when compared with missense mutations; however, among different groups studied so far, the results of the phenotype-genotype data have not been consistent (28). In our study, when the type of mutation was compared with the clinical features of the patient, no clear correlation was detected. In the next step, we examined the genotype/phenotype correlation in the group of patients with same *MECP2* mutation. For this, we studied the patients with recurring R306C mutation. The clinical symptoms were variable in three patients with R306C mutation. This shows no specific correlation between R306C mutation and a significant phenotype. Likewise, a previous genotype/phenotype correlation study did not offer any definite data in groups of patients showing the same mutation (29). Generally speaking, correlation of specific RTT mutations with a significant clinical manifestation can be hindered by the heterogeneity of this disease, as, even between patients with the same *MECP2* mutation, symptoms vary greatly (14).

**In conclusion,** mutations in *MECP2* are a common cause of Rett syndrome and mutations in *MECP2* gene can be found in the majority of Iranian RTT patients. Exon 4 of *MECP2* gene should be sequenced first. If no mutations are found, other exons should also be screened.
